# Army Veterinary Corps

**Published:** 1900-08

**Authors:** 


### EDITORIALS.

### ARMY VETERINARY CORPS.

In the June number of the Journal we published the discussion in the Senate of the United States on the adoption of Senator Kenney's amendment to the bill to increase the efficiency of the military establishment of the United States, which became Section 14 of said bill (commonly known as the Army Reorganization bill).[1]

Although this legislation had received an unusual amount of publicity, as we had been obliged to "educate" many otherwise intelligent people on this neglected question, it was remarkable how absolutely ignorant of the subject the few active opponents and their unthinking followers were.

For the latter an explanation is easily found in the following quotation:

"Congress is a body of small legislatures. The vast mass of affairs involved in the care of the government are subdivided, and the consideration of them is assigned to committees. On every subject, except those of political party importance or unquestioned merit, the action in regard to them is left to a committee to decide, and the vote in the Senate and the House of Representatives is a perfunctory measure.

"Fortunately for us, the *establishment of a Veterinary Corps in the Army is one of unquestioned merit*, and appeals to the common sense and first instinct of economy in every legislator who has taken the time to consider it.[2] All of the higher authorities in the army have expressed their opinion that its immediate establishment is a necessity."—Journal of Comparative Medicine and Veterinary Archives, January, 1900.

For the former, we can only credit them with not having studied the subject in order to understand it.

The main object of the bill (to make an organized effective service such as exists in the army of every other country in the

[1] See Congressional Record, May 4, 1900, and Journal of Comparative Medicine and Veterinary Archives, June 1900, page 361.

[2] We can safely state that two-thirds of the Senators who voted against the Veterinary Bill in the Senate had not given it personal attention, but voted "nay" because the Military Committee had not reported favorably upon it. One of them, in reply to a letter asking for his consideration of the Veterinary Bill, wrote back a civil letter stating his opinion on the "Loud" Bill, a post-office matter, which showed how little attention he had given it.

civilized world, and is approved by all the older officers of experience in the United States Army) was evaded by the opponents of the bill, and personal opinions and distorted facts were the only arguments advanced. The veterinary matter was not considered by the Senate Committee, and when a member of that committee requested a hearing upon it he was informed that it would "NOT BE CONSIDERED."

However, the Senate did consider it, and did so favorably.

Capital was made of the improved status of the veterinary service; "*an entire change since the civil war*," which consists in the provisions of the Act of March 2, 1899—thirty-five years after the civil war.

The following official rulings show the status referred to:

[CIRCULAR, NO. 55.]

HEADQUARTERS OF THE ARMY.
ADJUTANT GENERAL'S OFFICE,
WASHINGTON, NOVEMBER 23, 1899.

The following decisions have been made and are published to the Army for the information of all concerned:

2. STATUS AND ALLOWANCES OF VETERINARIANS. A veterinarian appointed under the Act of Congress approved March 2, 1899, is not a commissioned officer or an enlisted man, but a civil employé. A veterinarian of the second class is entitled to all the allowances or emoluments of a sergeant-major, other than his pay proper, which is fixed by law, the same as if he were an enlisted man.—[*Decision Sec. War, November* 7, 1889.--290576 *A. G. O.*]

By command of MAJOR-GENERAL MILES:

H. C. CORBIN,
*Adjutant General.*

RULINGS: PAYMASTER-GENERAL'S OFFICE.

VETERINARIANS: First class, pay $125 per month. Act approved March 2, 1899. G. O. 36, A. G. O., 1899.

*Note:* Under decision of Secretary of War, a veterinarian is a civil employé, and as such is not entitled to service pay, commutation of quarters, mileage, nor to travel pay upon discharge. Par. 2, Cir. 55, A. G. O., 1899, and Dec. 2d, Comp., par. 2087, edition of 1869.

Second class, pay $75 per month. Act approved March 2, 1899. G. O. 36, A. G. O., 1899.

*Note:* Authorized to charge $3.71 per month on monthly pay account, in lieu of clothing allowance. Letter Adjutant-General to Paymaster-General, dated January 27, 1900. Not entitled to increased pay for service, mileage, nor travel pay upon discharge. Same status with regard to civil employé as first class veterinarians.

The following testimony reviews the subject almost as completely as it can be gone into:

[EXTRACTS FROM HEARING BEFORE THE COMMITTEE OF MILITARY AFFAIRS OF THE HOUSE OF REPRESENTATIVES ON THE BILL (S. 4300) ENTITLED "AN ACT TO INCREASE THE EFFICIENCY OF THE MILITARY ESTABLISHMENT OF THE UNITED STATES."]

HOUSE OF REPRESENTATIVES,
MONDAY, MAY 14, 1900.

The committee met at 10.30 o'clock A.M., Hon. J. A. T. Hull in the chair.

*Statement of Dr. D. E. Salmon, Chief of the Bureau of Animal Industry, United States Department of Agriculture.*

The Chairman: Dr. Salmon, the committee would be glad to hear from you. I believe you are somewhat interested in some of the provisions of this bill.

Dr. Salmon: I would like to explain, Mr. Chairman, that I am not connected with the Army. I have no desires in that direction; but I come here to represent the American Veterinary Medical Association, which appointed me chairman of a committee to present the matter of veterinary service in the Army to Congress, with the object of getting a more efficient service than we have had in the past.

The veterinarians are interested in this matter for two reasons. In the first place, they are patriotic citizens of the country, and they have had an opportunity to know something of the veterinary service in other armies and in our Army, by reason of having had members of our association in the veterinary service of the United States; and in the second place, they desire to secure some amelioration of the condition of the veterinarians in the army service.

Our members who have been in the Army have presented to us the unpleasantness of their condition in the Army under the present system, the impossibility of improving the service, and the fact that men of self-respect can hardly remain there, to such a degree that the association has become interested in the matter, and desirous, if possible, to have Congress do something to give the veterinarians the status which is given to other educated men and the representatives of other professions.

Now, the veterinarians have had some opportunity to know what is required of veterinarians in the army service and how necessary their assistance is for the efficiency of the Army, because, really, the necessities of the European armies constituted the foundation of the veterinary profession. The first veterinary schools were established in France because of the needs of the military service, and the advantages of having trained veterinarians were so soon seen by other countries that veterinary schools (military veterinary schools, mostly) were established in the principal countries of Europe; and I believe every one of the European armies has an organized veterinary corps and an efficient veterinary service. The Army of the United States is, I think, the only one belonging to any of the civilized countries of the world which has not such a service organized upon a basis which will insure good men and a good, efficient service.

Now, our association feels that this provision, as it was passed by the Senate, meets the needs of the case, and it would like very much, if this committee could see its way clear, and thinks it advisable to do so, that this provision should be reported just as it passed the Senate, for the reason that if any changes are made there is a possibility not only that there may be nothing gained for the veterinary service at this session, but that we may get something that is not as good as the provisions which were passed by the Senate.

Our association has always held that there should be an organized veterinary corps, with a head to it. That is to say, we think that the veterinarians scattered through the cavalry regiments of the Army, with no coördination between them, with no expert director, cannot give really efficient service. The veterinarians who are provided for, and who are likely to be provided for, to go with the regiments must necessarily be men of a somewhat lower grade than the average members of the profession. That is to say, it is impossible to get men who have had experience, and who have been successful veterinarians, to take positions for $75 or $125 a month in the Army, even if they had rank; because that class of men are making from $2500 to $10,000 per year outside of the Army. But we feel that with a corps organization these men would be all right if there were at the head of them an experienced and successful veterinarian, and if a salary could be given which would command that kind of a man. Such a chief, whatever he might be called, would be able to give directions for the veterinary service; he would be able to see that the men under him were performing their duties; he would be able to see that they had proper supplies; and, in fact, he would be able to raise these men, approximately, to his level.

It would be just as it is in the meat-inspection service in the Department of Agriculture, of which I have the honor to be the head.

Now, the Government could not maintain a respectable meat-inspection service if it employed $900 or $1500 men and placed these men by themselves, isolated, without any coördination between them, one at each packing-house of the country. It takes all of my time and attention to see that the men in the different places have instructions, to see that they make their reports, to see that they are carrying on their work properly, and to hold them responsible for it. It appears to me, and it appears to the profession, that such an organization is necessary in the Army in order to get the proper service out of the men, in order to see that they are performing their duties uniformly and harmoniously throughout the different regiments and in the different places where they are placed, and in order to see that they are all kept up to the times in their work.

Those are the principal reasons why the veterinarians desire a veterinary corps.

The Chairman: Gentlemen, have you any questions to ask?

Mr. Marsh: Doctor, how many veterinary surgeons are there under your jurisdiction as the head of this service?

Dr. Salmon: In the inspection service of the Department of Agriculture?

Mr. Marsh: Yes.

Dr. Salmon: About 200.

Mr. Marsh: Are they commissioned?

Dr. Salmon: They are not commissioned, no, sir. They are not in the military organization.

Mr. Marsh: They are employés?

Dr. Salmon: They are employés, yes, sir; but they have an organization. They all report to me. They all get their instructions from me.

Mr. Marsh: To whom do you report?

Dr. Salmon: I report to the Secretary of Agriculture.[1]

[1] So should the chief veterinarian of the Army report to the Secretary of War, as he does in the armies of other countries.

Mr. Marsh: So far, there are, I think, eleven departments of the War Department, are there not?

Dr. Salmon: I do not know how many there are.

Mr. Marsh: And this is a proposition to make a new one?

Dr. Salmon: It is a proposition to organize the veterinarians into a corps; yes, sir,

Mr. Marsh: A staff corps?

Dr. Salmon: Yes, sir.

Mr. Marsh: There are now, I think, eleven staff corps in the Army, each independent of the other, and each reporting directly to the Secretary of War. This is a proposition to create another staff corps?

Dr. Salmon: Yes, sir.

Mr. Marsh: Some gentlemen desire to avoid that and at the same time provide for an efficient veterinarian service in the Army. Now, do you know of a way by which an efficient veterinary service can be organized in the Army without establishing a separate, distinct, and independent veterinary corps?

Dr. Salmon: No, sir; I have never discovered any way in which it can be done, and I do not think any other country has, because in every army of Europe, certainly in the armies of all the principal countries, there is a separate, independent veterinarian corps.

Mr. Marsh: Does the veterinarian corps report to the secretary of war, the head officer of the war department of the army?

Dr. Salmon: I think in most countries they report to the secretary of war. At any rate, I understand that in all those cases the veterinary organization is the same as the medical organization. The two services are practically parallel.

Mr. Marsh: We have now in the service of the War Department eleven staffs—the Adjutant-General, the Inspector-General, the Corps of Engineers, the Ordnance Department, the Quartermaster's Department, the Commissary Department, the Judge-Advocate's Department, the Signal Corps Department, the Record and Pension Office Department, the Pay Department, and the Medical Department. There is a feeling among some that those departments ought to be reduced in number instead of increased. Is there any one of the departments under which this service could be placed without increasing the number of staff departments?

Dr. Salmon: I do not know about that. The veterinarians would prefer to have the bill passed as it passed the Senate; and then when the Army reorganization bill comes up (as I understand there will be an Army reorganization bill next session), they will be willing to accept any organization which is applied to the Army as a whole. I think it would be quite important and advisable to have a similar organization for the different corps of the Army, and have them report somewhat in the same way; and then, if it is deemed best to change the organization in that respect, I do not think there would be any objection at all.

What we are most anxious for is to get some recognition for the veterinarians now, and get a service established on some kind of an efficient basis. I do not think it is material to the veterinarians whether the head of this corps reports to the Secretary of War or to some other officer, so long as they have a corps with a head to it—somebody who can give expert directions to the corps and give expert advice to the Secretary of War.

Mr. Marsh: Would you have the members of this corps subject to the orders of the head of the corps, as, for instance, the surgeons in the Army are subject to the orders of the medical corps—ordered here and there, attached to this command next month and to another command three months hence, and changed around from one place to another—instead of allowing them to be permanently attached to the regiments which have horses?

Dr. Salmon: I think that would be a matter of War Department detail, and a detail of administration upon which I should not care to express any opinion. I have not been in the service, and I do not know what would be best in that case.

Mr. Marsh: Under the present system, the Surgeon-General orders Surgeon So-and-so to Fort Worth, Tex., to-morrow, and orders the surgeon already there to some other place, and is continually changing them around from one place to another. They are on duty in the west first in one department and then in another, so that they are not with any degree of permanence attached to any one command. Under the present bill the head of this veterinary corps would have that very power. Do you think that would be a good condition of things?

Dr. Salmon: Well, I should think that if it were best for the medical department, it would be for the veterinary department; although, as I say, I have not had experience in the Army, and I should not care to express an opinion further than that. If it were proved by practice to be the best plan for the management of the medical department of the Army, I do not see why it should not be for the veterinary department; because the services are, in my opinion, parallel and comparable in every respect. Some one certainly should have the authority to take a veterinarian, in case of an emergency, from one regiment and put him in another, or to detail him to any service that might be necessary.

Mr. Marsh: That power exists now in the Secretary of War.

Dr. Salmon: Yes. I suppose the idea of the bill is to relieve the Secretary of War from details of that character.

Mr. Marsh: In other words, to take them out of his hands?

Dr. Salmon: No, sir; he would direct the chief of the veterinary corps, just as he directs the chief of the other corps.

The Chairman: As a matter of administration, the Secretary of War has control of the whole thing now?

Dr. Salmon: Yes, sir.

The Chairman: Of the Medical Corps and all other corps?

Dr. Salmon: Yes.

The Chairman: And he would have under this bill?

Mr. Marsh: Theoretically he has, and practically he has not.

The Chairman: I think he has absolute control, but he does not want to meddle with the matter.

Mr. Marsh: He has the power, but practically does not use it. The heads of departments do it.

Dr. Salmon: I know that the head of the Department of Agriculture prefers to have me take such details in the Bureau of Animal Industry and manage them, unless some special case comes up, a case involving the policy of the Department. In such cases, of course, I always go to the Secretary of Agriculture. So I should suppose the chief of a corps of this kind would always go to the Secretary in case there were a question of policy involved.

In regard to the mere details of stationing men at different points, and determining whether they are needed or not, and giving them veterinary instructions, I should suppose the Secretary of War would desire to be relieved of all such details.

The Chairman: Are there any other questions? If not, that is all, Doctor.

Dr. Salmon: I would like to add, if you please, Mr. Chairman, that there are some reasons why there should be veterinary advice for the War Department here in Washington. For instance, in conducting the examination for veterinarians in the past, and those which have been held recently, an expert has been detailed from the Bureau of Animal Industry to draw up the examination papers, and to inspect the papers after the examinations were held. I presume there are some questions of that kind constantly coming up in the War Department here which require veterinary advice, and the Secretary of War could apply to the head of the corps of this kind for expert advice at any time.

*Statement of Col. Rush S. Huidekoper.*

The Chairman: Colonel Huidekoper, the bill which has passed the Senate, establishing a veterinary corps, is now before this committee; and as you are something of an expert in the line of veterinary science, and have taken a good deal of interest in the consideration of this question, the committee would be glad to hear from you in regard to your views as to the advisability of passing the bill, changing it, amending it, enlarging it, or contracting it.

Colonel Huidekoper: Mr. Chairman, if you will allow me for just a moment, I will take up a very brief matter of history at the start.

In regard to veterinary medicine, this country is a full century behind all the European countries, from an agricultural standpoint. While all the countries in Europe started schools of veterinary medicine a century ago, most of them at the outset attached them to the ministry of war, that of Austria still being attached to that department, as was, originally, that of Italy; but to-day the schools of Italy and France are attached to the ministry of agriculture.

It was not until pleuro-pneumonia came to this country and increased in the large stock yards and dairy farms of Brooklyn, Long Island, and New York, and extended over into Pennsylvania and Maryland, in the late sixties and seventies, that any great attention was called to the need of Government veterinarians. There were so few of them here that the first general inspection of the condition of contagious pleuro-pneumonia and other diseases of animals required bringing Professor Gamgee, an Englishman, to this country for that purpose.

It was about that time, or later, when the pleuro-pneumonia spread on to Chicago and into Kentucky, that we saw the necessity for properly educated men of a class different from those we had in this country. We had a few men who had come from foreign schools; we had a few hundred men (among them some of the best we had) who were self-educated; but they were individuals, and exceptions.

About that time we saw the necessity, from an agricultural stand-point, of better educated men. Two large land-owners and farmers in Pennsylvania, together with Mr. J. B. Lippincott, the book publisher, of Philadelphia, who was president of the Humane Society of that city, wished, from a humanitarian point of view, to establish a school for this purpose, and they erected a building in the University of Pennsylvania. I was at that time very much interested in animals, while practising medicine, and I spent a couple of years abroad looking the subject up, as a result of which this school was established on a basis equal to that of the medical school. The same scientific education was given, and the same elementary education (covering chemistry, physiology, and anatomy) as is given in a medical school, together with a veterinary education equal to that given by the schools of Europe.

Almost at the same time Harvard University established such a school, and later New York State gave some $400,000 to Cornell University, at Ithaca, for the establishment of a school on the same basis. These steps have forced smaller private schools throughout the country to come up to a three-year standard of education, and it is those schools which have furnished the men who are now employed by the Bureau of Animal Industry, as well as the better class of veterinarians throughout the country.

A few years after the establishment of the school at the University of Pennsylvania General Sheridan, who had just returned from his long inspection of the armies of Europe (you remember he made a long visit in Europe, looking over the armies there), happened to be in Philadelphia, became interested in this subject, and invited me down here to Washington to go over the subject with him, as he had seen the veterinary service of the armies of Europe. At that time Col. Sanford Kellogg and I drafted a bill or an outline for an organization modelled after European organizations, which General Sheridan corrected, and expected to have recommended at the next session of Congress, when his death prevented that being done. General Schofield the following year took far less interest in the matter than General Sheridan had taken, and, as you know, you have for years had before this committee some feeble attempts at legislative improvement of the veterinarians in the Army until last year, when more active steps were taken.

The present bill provides for a smaller corps than that wished for by General Sheridan. Last winter, when the Army bill, known as the Hull bill, was in the House here, I happened to be in Washington and in consultation with Dr. Salmon, and we took up the subject again and carried it to the President, by whom it was referred to the Secretary of War, who told me to return in twenty-four hours. At that time I placed in his hands the data regarding European armies, and the condition of the veterinary service in our own Army; and at the end of twenty-four hours Secretary Alger indorsed an amendment to be submitted to the Senate, which was practically the same as that which we are asking this year. As the provisional bill took the place of the regular bill, no action was taken on that amendment, and our association returned again this year with the same amendment, calling for the establishment of an organized service.

The present condition of the veterinarians employed in the Army is that of a mere civilian employé. You will notice that the pay of veterinarians of the second class is $75 a month, with the allowances of a sergeant-major, which allowances include clothing and rations. A sergeant-major can get commutation of rations; and you will remember that when you passed the appropriation bill a few weeks ago it required a proviso for commutation of clothing of $3.75 a month to these men, because while they have the allowances of a sergeant-major they do not wear a uniform, and therefore the allowance of clothing was useless to them, and yet there was no provision for commuting their clothing. The Commissary-General, at about the same time, had to issue a similar order for the commutation of rations.

Again, the Quartermaster's Department employs an irregular number of men—some twenty or thirty—who were called in for ordinary, small services. They, again, are mere civilians, under a quartermaster, reporting to no one except the officer to whom they are directly assigned for duty, and for services which are purely of a practical nature.

The services performed by the veterinary surgeon employed by our Army to-day consist in his being called in to treat sickness after it occurs, to treat lameness after it occurs, and to do small detail duty. He does not perform any of the functions which exist in foreign armies, and which form the principal part of the service of those armies. The War Department in its examinations for promotion to the staff departments of the Army gives one number out of thirty for hippology, and yet the officers have had no instruction in it beyond their practical experience. As our officers leave West Point to-day, young cadets come out as second lieutenants, to take command of a troop of cavalry or a light battery of artillery, the senior officers having been promoted or placed in command of larger bodies of men, and unless these young men have been horsemen, or the sons of horsemen, before going to West Point, they know nothing about a horse, his shoeing or his stable care; they have had no instructions of any kind in hippology. In every military school in Europe there is a thorough course of instruction to the cadets and the younger officers on the conformation of the horse, the anatomy of the foot, shoeing, stable hygiene, grooming, the character of forage, the character of oats and of hay, which is as much instruction, both theoretical and practical, as can be given to a layman for the purpose of making him a good horseman. It is, in reality, such a course of instruction as is followed by many a civilian who owns a large stock farm.

At our military schools at Riley and Leavenworth there is some slight instruction given, but nothing which nearly equals the instruction which is given in every school in Europe. There the farriers, the horseshoers, and the first sergeants receive instructions at the military schools from a competent veterinarian on those same subjects—stable hygiene, grooming, washing, and cleaning the horse's sheath, the anatomy of the foot, shoeing, packing with saddle or pack, the points of bearing of a harness, and so forth, so much as can be given to make them competent men, and also instruction in the treatment of ordinary diseases.

Take the French army as an example. In that army every first sergeant, and horseshoer, and farrier of a troop is capable of doing about the same work that we ask to-day of the veterinarians employed in the Army. They cannot have a large number of veterinary surgeons in every regiment. They have two to a regiment to do those duties which the regimental commanders require of them, and besides what they do themselves to see that the other men perform those details of work which belong to the practice of veterinary medicine.

When a troop leaves, every troop carries one pack-horse, with a small outfit of instruments and medicines, and instruments for immediate ordinary use by the blacksmith and farrier, far better than we supply in our Army for the use of the veterinarians. As I will show you by the tables, the quartermaster furnishes to the veterinary surgeon of a French regiment enough supplies for all ordinary emergencies; and a man who has received a proper amount of training is able to supervise the work of the other men, to look after the general hygienic condition of the stables, to act as an aide to the colonel of the regiment, and to make such a special inspection as belongs to him; and has, in addition, time for the inspection of the animals that are to be slaughtered for food for the regiment. I am advised by all with whom I have consulted—General Miles, General Brooke, General Merritt, General Wilson, the Quartermaster-General, the Commandant at West Point—that such a course of instruction should be inaugurated at West Point, Fort Riley, and Fort Leavenworth the instant we can get the men for it.

On the question of boards of purchase let me say this: a horseman is born; it comes natural to him. Some men are naturally horsemen, and some men will never be horsemen, no matter how much they study. An old officer, a cavalry or an artillery officer, who has handled horses, or a quartermaster who has a great deal to do with the handling of horses and mules, knows what a good draft animal is. An old artilleryman knows what character of animal, as to conformation and quality, he wants for an artillery horse, or what is wanted for hauling ambulances in the medical department, and a cavalry officer what class of saddle horse is wanted for the cavalry; and these men are perhaps the best judges, just as a good horse-dealer is often the best judge of a horse that exists. And these men are the proper ones to purchase horses in large numbers, but they are not experts on the details of soundness. If an apparently sound horse is bought who has had one or two attacks of "moon-blindness," and two or three months afterward he goes blind on their hands, it shows the necessity for some other system of purchase. Take the matter of defects of a hock or of a pastern of the forefoot; such things look well enough in the general conformation of the animal, but an expert who knows every bone of the joint can run his hands over them and say at once whether a spavin is started, or will come out, or whether a ringbone is starting in the forefoot. It requires men of that class to take a lot of feet with more or less defects and determine whether they are defects of shoeing or are pathological conditions which will become permanent and end in uselessness.

The veterinary member of a board of purchase is simply an auxiliary member. The officer, the cavalry or artillery officer, is the man who knows the character and quality of the horse he wants purchased; but the veterinarian attached to the board is the man who can best determine its physical condition.

For the last seven years the Government of France has kept in this country a board of five officers, consisting of three line officers and two veterinary surgeons, for the purpose of purchasing horses. They have been all over Ohio, Indiana, and throughout the entire West, and they have been here for seven years. During that period Germany has had one or two boards here for some time. During the entire year previous to the American-Spanish war Spain had in the United States a board of three officers, two line officers and one veterinary surgeon, purchasing mules for Cuba; and our own animals likewise should be purchased by men who are competent to know what a good horse is, and who can determine that the animals selected have no internal defects.

There are many internal diseases, such as heart disease or lung disease, for which every horse ought to be examined. Many a horse is apparently good through ordinary tests and exercises, until he is put to hard work——

The Chairman: I doubt if we have time for this whole description, Colonel.

Colonel Huidekoper: I am practically through.

The Chairman: What we want to get at is the question of organization.

Colonel Huidekoper: Now, the question of an effective organization is a business proposition. It is identical with the case of anyone who has a large farm with a number of buildings on it, or any other business where twenty, thirty, or fifty workmen are employed, and the work has to be more or less harmonious. A man in such a business employs a foreman to look after the different matters, and see that the work is well done, unless he can look after it himself. I cannot suggest any other plan for the accomplishment of this object than such an organization as exists in Europe, where the veterinarians are given a separate corps. One thing which seems to be a good deal of a bugbear, which has been used by the few opponents of this plan, is this very matter of a separate bureau, which, from a business point of view, is nothing but an organization to insure effective work. That is instanced by the fact that one of the last bureaus, that of Pension and Records, could have done nothing without a separate head.

As you will see, this bill has been so drawn as to bring out men absolutely by competitive examination. From the time of the appointment of the first twenty to supply the ten regiments of cavalry, you will see that the promotion to the next grade, that of first lieutenant, is to be after a competitive, satisfactory examination. There is absolutely no question of appointment except on the merits of the men, and that fact gives them something to work for. That would give ten competent men for the Light Artillery, the Quartermaster's Department, and for the supervision of the inspection of meat for the Commissary Department.

Again, at the end of three years, four of these veterinarians are, by "competitive, satisfactory examination," to be selected from the ten assistant veterinarians, and given the rank, pay, and allowances of a captain of cavalry. These men would then be competent, I believe, to go to West Point, Fort Riley, or Fort Leavenworth as teachers, or to do expert work where required.

Mr. Marsh: Let me ask you there, Colonel, whether you would have the veterinarians examine the meat furnished by the Commissary Department, or whether you would have the surgeons of the Army examine it? Do you understand that to be one of the duties of a veterinarian?

Colonel Huidenkoper: It is in all the European armies.

Mr. Marsh: Is that what you want introduced into this Army?

Colonel Huidekoper: At a future time; yes, sir.

Mr. Marsh: That the veterinarians shall examine the meat which is dealt out to the men, or that the surgeons shall do it?

Colonel Huidekoper: The examination of live stock and quartered meat belongs properly to the veterinarian as an expert. He is the one who has studied the diseases to which the animals are subject, and is a better expert in regard to them than a medical can be.[1]

(To be continued.)

[1] In Europe all animal food for the soldiers is inspected on the hoof and when dressed by the army veterinarians before it is issued as rations. Our army depends upon a chance of the meat food coming from a packing-house which employs veterinarians of the Bureau of Animal Industry. There is not to-day a qualified meat-inspector in the United States.

A commissary or other officer may know the difference between spoiled meat and fresh, but he, and even the medical officer, is not familiar with the tape-worm, the trichinæ, the lesions of tuberculosis or other diseases as they occur in animals, rendering them unfit for food.—*Troy Times, April* 18, 1900.

